# Efficacy of Shen Cao Gan Jiang Tang on Non-severe COVID-19 Patients: A Multicenter, Randomized, Open-Label Clinical Trial

**DOI:** 10.7759/cureus.62380

**Published:** 2024-06-14

**Authors:** Dieu-Thuong Thi Trinh, An Hoa Tran, Minh-Man Pham Bui, Thy Xuan Kieu, Van-Dan Nguyen, Nguyen Huu Lac Thuy, Khac-Minh Thai, Nguyen Lam Vuong

**Affiliations:** 1 Traditional Medicine Administration, Vietnam Ministry of Health, Hanoi, VNM; 2 Faculty of Traditional Medicine, University of Medicine and Pharmacy at Ho Chi Minh City, Ho Chi Minh, VNM; 3 University Medical Center at Ho Chi Minh City, Branch 3, University of Medicine and Pharmacy at Ho Chi Minh City, Ho Chi Minh, VNM; 4 Faculty of Pharmacy, University of Medicine and Pharmacy at Ho Chi Minh City, Ho Chi Minh, VNM; 5 Department of Medical Statistics and Informatics, Faculty of Public Health, University of Medicine and Pharmacy at Ho Chi Minh City, Ho Chi Minh, VNM

**Keywords:** sars-cov-2, covid-19, herbal medicine, traditional medicine, shen cao gan jiang tang

## Abstract

Background

In the face of the escalating COVID-19 pandemic amid shortages of medications and vaccines, a Vietnamese herbal formula known as Shen Cao Gan Jiang Tang (SCGJT) has been put into use for non-severe COVID-19 patients. This study aims to assess its efficacy and safety.

Methods

A multicenter, open-label, randomized controlled trial was conducted on 300 patients with non-severe COVID-19, randomly assigned into two groups: 150 receiving standard care (control group) and 150 receiving additional SCGJT for 10 days (SCGJT group). Time to resolution of symptoms, symptom severity, disease progression, time to discharge, the National Early Warning Score 2 (NEWS2) score, usage of Western drugs, time to viral clearance, and safety outcomes were continuously monitored.

Results

The SCGJT group exhibited faster symptom resolution (median: 9 vs. 13 days) and improved symptom severity, including cough, fatigue, hypogeusia, muscle aches, nasal congestion, runny nose, and sore throat, compared to the control group. Although there was a lower rate of severe progression in the SCGJT group (0.7% vs. 4.7%), the difference was not statistically significant. The time to discharge was significantly shorter in the SCGJT group (median: 7 vs. 8 days). Changes in the NEWS2 score did not show significant differences between groups. SCGJT has been demonstrated to reduce the need for symptomatic relief medications and hasten SARS-CoV-2 viral clearance. No adverse events were reported, and routine tests showed no significant differences.

Conclusions

SCGJT is safe and has potential clinical efficacy in non-severe COVID-19 patients. However, data regarding preventing severe progression remains inconclusive. Further studies should be conducted in light of the current state of the COVID-19 pandemic.

## Introduction

The COVID-19 pandemic has presented unprecedented challenges to global healthcare systems, particularly impacting low- to middle-income countries (LMICs) by impeding their access to vaccines and medicines [1]. LMICs have encountered significant challenges in effectively procuring and administering conventional treatments due to limited production capacities, global distribution disparities, financial constraints, and logistical hurdles [1]. Emphasizing the importance of accessible and culturally relevant healthcare options, the investigation of traditional medicine (TM) drugs holds significant potential for supplementing the treatment options available to LMICs during the COVID-19 pandemic [2]. TM has also demonstrated its utility in the management of prior viral diseases such as severe acute respiratory syndrome (SARS) and H1N1 influenza [3].

Following TM theory, COVID-19 is classified as a “pestilence,” characterized by its contagious nature and potential to induce pandemics. Wei Qi, or defensive Qi, protects the body from external pathogens and functions similarly to the immune system. In Shanghan Lun, a TM classic book written by Zhang Zhongjing, a Taiyang exterior condition is regarded as Wei Qi. In the early phase (Taiyang syndrome), patients exhibit clinical symptoms such as fever, muscle aches, cough, sore throat, and other systemic symptoms. Therefore, in the context of treating COVID-19, TM emphasizes specific principles aimed at fortifying Qi, bolstering defensive Qi, and restoring Qi equilibrium to shield the body from the invasion of external pathogens [4,5].

Shen Cao Gan Jiang Tang (SCGJT) has been employed in the treatment of COVID-19 in Vietnam alongside other formulas such as Shen Su Yin, Renshen Baidu Tang, and Bu Fei Tang. It is a modified traditional remedy composed of *Radix Glycyrrhizae*, *Rhizoma Zingiberis*, and *Radix Ginseng*, derived from Gancao Ganjiang Tang formula in Shanghan Lun, for recuperating depleted Yang (Taiyang syndrome) with some main symptoms such as aversion to cold, dyspnea, and fatigue [6]. In Shanghan Lun, Zhang Zhongjing often combined ginseng to increase the effect of nourishing Wei Qi and eliminating external pathogens in Taiyang syndrome. Therefore, SCGJT can help nourish Qi, aiding the body in resisting external pathogens in the early phase (Taiyang syndrome). In addition, *Radix Glycyrrhizae *and *Radix Ginseng*, which demonstrated effectiveness in SARS treatment during the 2002-2003 epidemic, are considered to have a relatively high priority for immediate investigations on their potential usefulness in the treatment or prevention of COVID-19, based on available toxicological information [7].

Since SCGJT was widely implemented in Vietnam, a pilot study with unpublished data has preliminarily indicated efficacy and safety in non-severe COVID-19 patients. However, the application of SCGJT in the treatment of COVID-19 patients still lacks sufficient clinical evidence. In this study, the efficacy of SCGJT in reducing symptoms, clearing the virus, and improving prognosis in non-severe COVID-19 patients, as well as its safety, were assessed.

## Materials and methods

Trial design

This was a multicenter, balanced randomization (1:1), open-label, parallel-group study aimed at evaluating the efficacy and safety of the SCGJT herbal formula in treating non-severe COVID-19 patients. The study was conducted from September to December 2021 at five COVID-19 isolation and treatment centers, including the University Medical Center Ho Chi Minh City, Tân Bình COVID-19 Field Hospital, COVID-19 Field Hospital No. 3, Thu Duc 2 Field Hospital, and the Centralized Quarantine Facility at Thu Thiem College.

The study was carried out in accordance with the ethical principles outlined in the Declaration of Helsinki and the International Conference on Harmonization-Good Clinical Practice guidelines, as well as the Consolidated Standards of Reporting Trials (CONSORT) [8]. This research was reviewed and approved by the institutional review board of the University of Medicine and Pharmacy at Ho Chi Minh City (approval number 458/HĐĐĐ-ĐHYD, dated August 20, 2021). Informed consent was obtained from all participants.

There were a total of 12 doctors who participated in screening subjects and administering treatments. All doctors underwent training and adhered to the research protocol for one week before conducting the study, as standardized by the research team to achieve standardization across multiple centers.

The study protocol was registered on ClinicalTrials.gov under the identifier NCT05055427.

Participants

The study recruited non-severe COVID-19 inpatients, with the following inclusion criteria: (i) aged between 18 and 64 years; (ii) confirmed SARS-CoV-2 infection by a positive reverse transcription-polymerase chain reaction (RT-PCR) with a cycle threshold (CT) <30; (iii) classified as mild or moderate COVID-19 according to the criteria set by WHO severity definitions [9], with symptom onset not exceeding five and 10 days, respectively; and (iv) not using any other herbal medicines during participation in the study.

Exclusion criteria were as follows: (i) presence of any uncontrolled medical conditions or conditions that could potentially interfere with the intervention and outcome assessment, including chronic obstructive pulmonary disease, kidney failure requiring dialysis or creatinine ≥2.0 mg/dL based on medical history, parenteral or enteral nutrition; (ii) presence of severe medical conditions, including cancer patients undergoing chemotherapy or radiotherapy, compromised immune systems like those with AIDS, recipients of anti-rejection medications post-transplant, immunosuppressive diseases, or patients with severe chronic illnesses, can potentially impact the treatment outcomes of COVID-19 according to researchers’ assessments or with psychiatric disorders or cannot take medicine; (iii) known allergy to products containing ginseng; (iv) pregnancy or lactation; and (v) taking part in other current intervention studies.

Randomization and blinding

Eligible participants were allocated to either the SCGJT or the control group. Block randomization was employed using SAS software version 9.4, with a block size of four. Randomization codes were placed inside envelopes with sequentially ordered numbers. Participants meeting the eligibility criteria were assigned to receive either SCGJT or standard treatment in accordance with the recruitment sequence. This was an open-label trial without masking because of the depletion of healthcare resources caused by the rapid increase in cases at the time of the study.

Interventions

Both groups received standard care in accordance with WHO guidelines [9], which encompassed standard nutrition, adequate hydration, and the administration of symptomatic treatments such as analgesics, antipyretics, antitussives, and antidiarrheal medications when deemed necessary.

Since randomization, the SCGJT group has received an additional dose of SCGJT decoction equivalent to two sachets per day, administered at 12-hour intervals continuously for a duration of 10 days. Each sachet contained 90 ml of liquid, including 6 grams of honey-fried *Radix Glycyrrhizae*, 3 grams of stir-baked *Rhizoma Zingiberis*, and 3 grams of *Radix Ginseng*. This dosage was determined based on the typical dosage of herbs, which had been observed to have no acute toxicity and showed no unusual signs in terms of weight, blood-forming, liver, kidney, and other organ function, except for a reduction in platelet count in male mice during semi-permanent evaluation in *Swiss albino *mice [10]. These herbs were provided by the Mediplantex National Pharmaceutical Joint Stock Company (Hanoi, Vietnam) with identical batches that underwent testing to ensure compliance with the criteria outlined in the Vietnamese Pharmacopoeia V (not less than 2.0% of glycyrrhizic acid in licorice, not less than 0.3% of the total amount of ginsenoside Rg1 and ginsenoside Re, and not less than 0.2% of ginsenoside Rb1 in ginseng).

Outcomes

The primary outcomes of the study comprised time to symptom resolution, symptom severity, disease progression, including severe COVID-19 and mortality, time to discharge, and the National Early Warning Score 2 (NEWS2) score.

Twenty-five symptoms were monitored, including anorexia, anxiety, aversion to colds, blood in sputum, chest pain, cough, diarrhea, dizziness, dry mouth, dyspnea, fatigue, fever, headache, hypogeusia, hyposmia, indigestion, insomnia, muscle aches, nausea, nasal congestion, runny nose, sore throat, spontaneous sweating, sputum production, and wheezing. Each symptom was assessed by participants on a four-point scale: 0 for no symptom, 1 for mild symptom, 2 for moderate symptom, and 3 for severe symptom. The severity of each symptom was assessed by the total score of that symptom from randomization until the symptom resolved, or for a maximum of 21 days. The global severity of all symptoms was determined by the total score of the 25 symptoms. Time to symptom resolution was defined as the duration from randomization to when the global symptom score reached 0. Severe COVID-19 was diagnosed according to the guidelines of WHO [9]. Mortality was determined when the hospital’s medical board concluded that the death was related to COVID-19. Patients were considered eligible for discharge if they were afebrile for at least two days, had stable vital signs, and obtained a negative RT-PCR result or a CT value greater than 30. The NEWS2 score was calculated based on vital sign values by investigators at the following website: https://ebmcalc.com/NEWS.htm

Secondary outcomes included time to viral clearance, utilization of Western drugs, and safety. Time to viral clearance was defined as the number of days from randomization to a negative RT-PCR result (CT >37) [11]. RT-PCR tests were conducted by collecting nasopharyngeal swabs on the fifth day following randomization and subsequently on a daily basis until a negative result was obtained. Safety was evaluated by monitoring the changes in routine lab tests and adverse events (AEs). Serious AEs were recorded when they resulted in severe consequences such as death, life-threatening conditions, hospitalization (initial or prolonged), disability or permanent damage, or interventions required to prevent permanent impairment or damage.

All outcomes were monitored from randomization until 21 days, except for the NEWS2 score, which was only monitored during the hospitalization period, and routine lab tests after 10 days of treatment. For patients who developed severe COVID-19, all outcomes, except for mortality, were discontinued.

Sample size calculation

The sample size was calculated based on detecting differences between the control and intervention groups in primary outcomes, with an alpha level of 0.05 and a beta level of 0.1. Previous studies reported that the average time to resolution of COVID-19 symptoms was 10 days in the control group and seven days in the TM group, with SDs of 5 and 3.5, respectively [12-14]. Therefore, a minimum sample size of 44 participants was determined for each group. For the progression to severe COVID-19, an estimated 1% incidence was expected in the TM group compared to 10% in the control group [14,15]. Thus, a sample size of 134 participants was required for each group. The largest calculated sample size of at least 134 participants was chosen, considering an anticipated 10% dropout rate, resulting in a minimum sample size of 150 participants in each group.

Statistical analysis

The efficacy analyses were performed using an intention-to-treat (ITT) dataset, comprising all patients who were randomized into the study, regardless of their adherence to the protocol or continued participation. Patients were analyzed based on the assigned study arm, maintaining the original randomization. Conversely, the safety analysis utilized the as-treated dataset, which included all patients who received at least one dose of the study drugs. Patients were analyzed according to the treatment group they actually received.

To illustrate time to symptom resolution and viral clearance, Kaplan-Meier plots were employed, with a log-rank test used to compare these measures between groups. Disease progression, utilization of Western drugs, and the achievement of negative RT-PCR results, as well as other categorical variables, were assessed for differences using Fisher’s exact test because some data did not meet the assumptions of the chi-square test, such as minimum expected frequencies over 5 for all cells or non-uniform data distribution. In addition, the NEWS2 score, time to discharge, symptom resolution and viral clearance, symptom severity, laboratory results, and other numeric variables were compared between the two groups using the Wilcoxon Mann-Whitney U test due to the non-normal distribution of the data. All statistical analyses were done using R version 4.1.0. All tests were two sided, and the significance level was set at 0.05.

## Results

**Figure 1:**
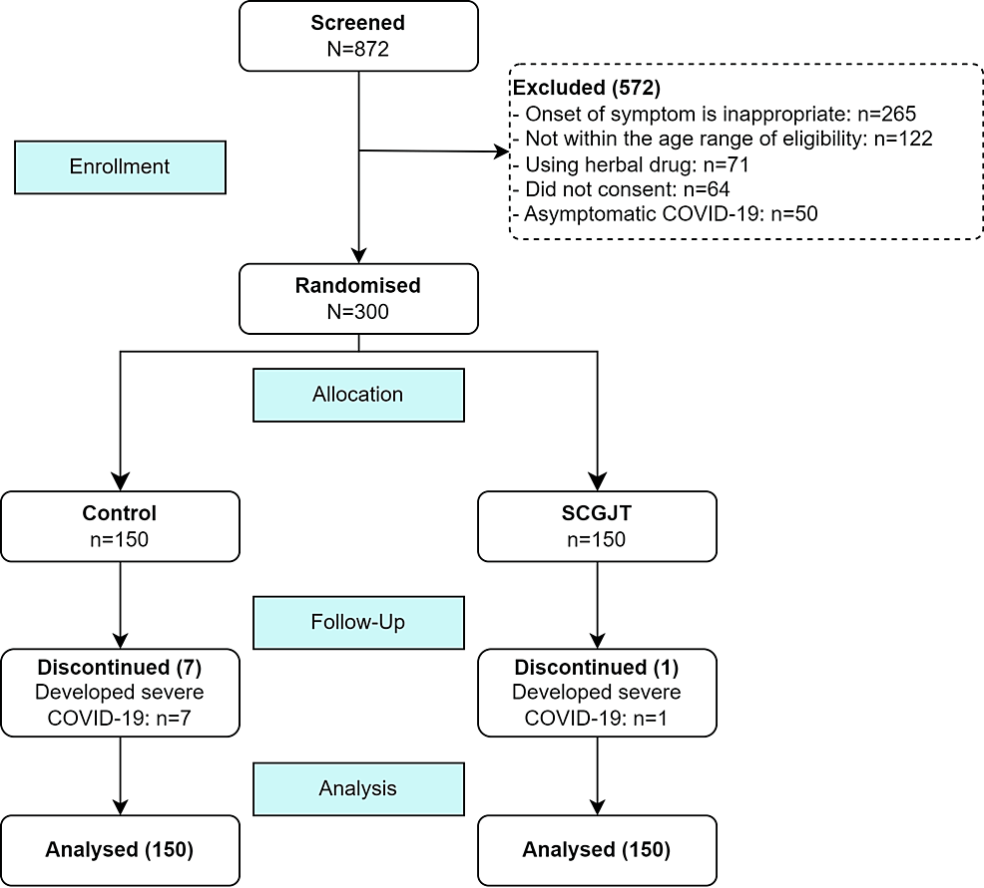
Flow diagram SCGJT, Shen Cao Gan Jiang Tang

From September to December 2021, a total of 872 patients were screened, of whom 300 were eligible for randomization. Seven patients in the control group and one patient in the SCGJT group developed severe COVID-19 and discontinued follow-up due to transfer to specialized treatment centers, excluding the mortality outcome. These eight patients were still included in the analysis according to the ITT principle and included up to the point of discontinuation (occurrence of primary outcome) (Figure 1).

Both groups demonstrated a balanced sex distribution with comparable mean ages (control: 42.8 ± 13.8 years, SCGJT: 42.9 ± 12.7 years). The duration from symptom onset to enrollment, prevalence of obesity, hypertension, and diabetes, along with COVID-19 vaccination rates, exhibited no notable disparities between the groups. Baseline clinical parameters, including the NEWS2 score, symptom score, CT value, and routine lab tests, were consistent, indicating a well-balanced distribution of demographic and clinical factors in both groups (Table 1).

**Table 1:** Baseline characteristics Note: Summary statistics are median (25th and 75th percentiles) or mean ± SD, or n (%). CT, cycle threshold; NEWS2, National Early Warning Score 2; SCGJT, Shen Cao Gan Jiang Tang

Characteristics	Control (N = 150)	SCGJT (N = 150)
Sex: male	74 (49.3%)	77 (51.3%)
Age, years	42.8 ± 13.8	42.9 ± 12.7
BMI, kg/m^2^	22.6 ± 3.1	22.8 ± 3.4
Days from symptom onset to enrollment	4 (3; 6)	4 (3; 6)
Obesity	31 (20.7%)	29 (19.3%)
Hypertension	28 (18.7%)	27 (18.0%)
Diabetes	11 (7.3%)	10 (6.7%)
Cardiovascular diseases	0 (0.0%)	4 (2.7%)
Pulmonary diseases	1 (0.7%)	2 (1.3%)
Thyroid diseases	0 (0.0%)	2 (1.3%)
Other comorbidities	5 (3.3%)	9 (6.0%)
Received vaccine for COVID-19		
None	24 (16.0%)	26 (17.3%)
One dose within two weeks	20 (13.3%)	25 (16.7%)
One dose for two weeks or more	106 (70.7%)	99 (66.0%)
NEWS2 score at enrollment	1 (0; 3)	1 (0; 3)
Symptom score at enrollment	13 (9; 18)	13 (9; 18)
CT value at enrollment	19.7 ± 3.7	19.6 ± 3.7
White blood cell count, ×10^9^/L	7.7 ± 2.1	7.5 ± 2.0
Neutrophil count, ×10^9^/L	3.8 ± 1.2	3.8 ± 1.3
Lymphocyte count, ×10^9^/L	2.5 ± 0.9	2.6 ± 1.0
Hemoglobin (g/dL)	12.8 ± 0.9	12.7 ± 0.9
Platelet count, ×10^9^/L	232 ± 57	233 ± 52
Aspartate aminotransferase, U/L	38.4 ± 14.4	37.6 ± 14.6
Alanine aminotransferase, U/L	37.3 ± 14.4	36.7 ± 14.3
Serum creatinine, umol/L	73.4 ± 19.9	74.7 ± 19.4
Fasting glucose (mmol/L)	5.8 ± 0.7	5.8 ± 0.7

**Figure 2:**
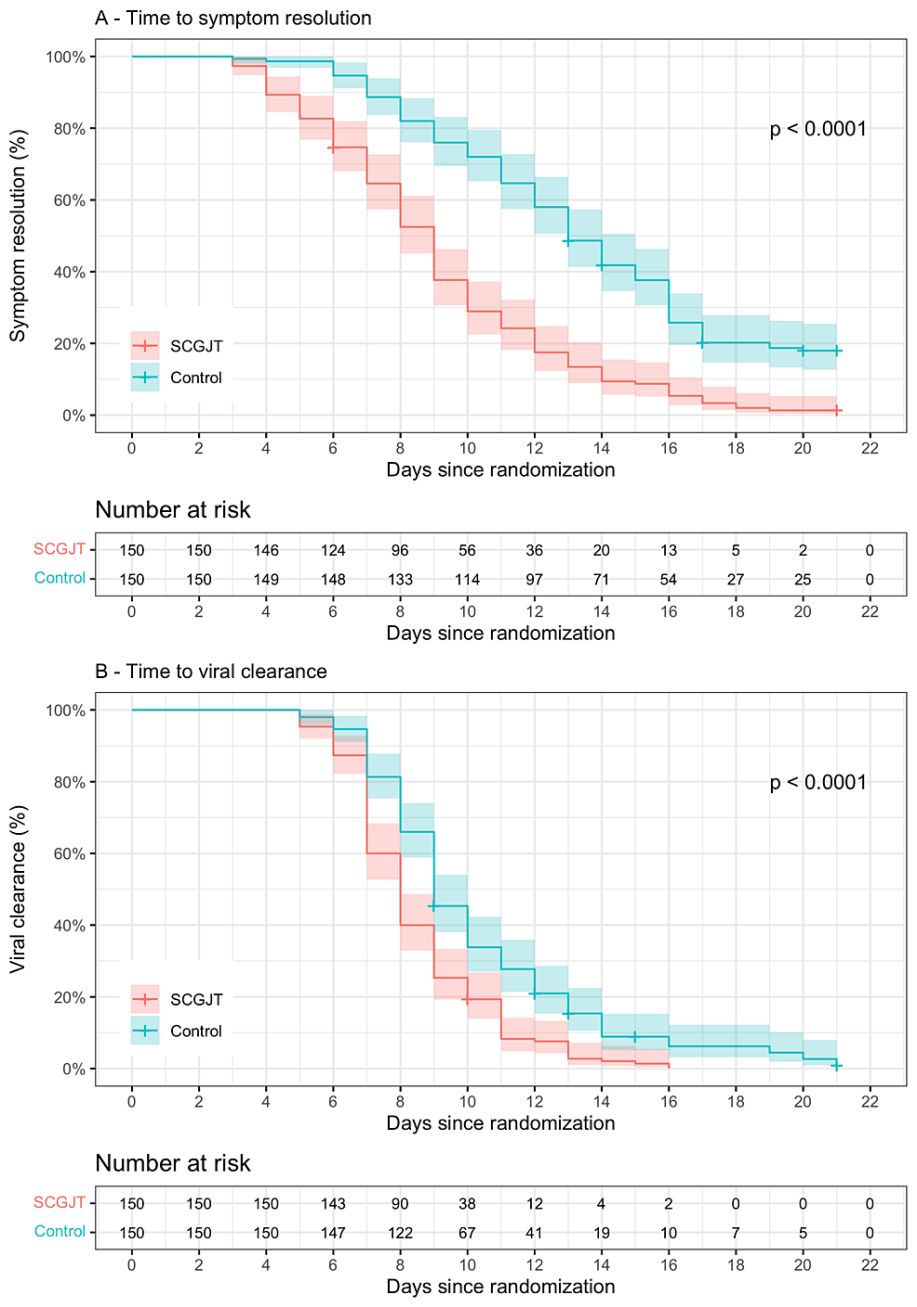
Time to symptom resolution and viral clearance SCGJT, Shen Cao Gan Jiang Tang

**Figure 3:**
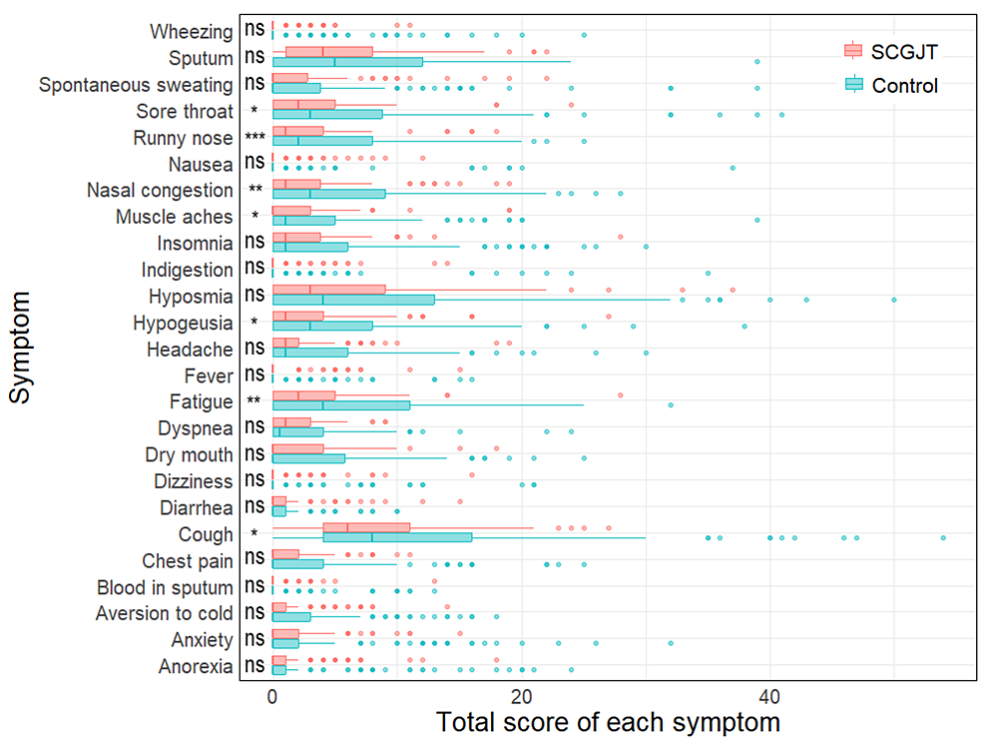
Total score of each symptom ns, nonsignificant; *p < 0.05; **p < 0.01; ***p < 0.001 SCGJT, Shen Cao Gan Jiang Tang

Time to symptom resolution in the SCGJT group was consistently lower than in the control group starting from day 4 since randomization (Figure 2A), with a median (25th and 75th percentiles) of 9 (6; 11) days compared to 13 (10; 16) days (p < 0.05). The SCGJT group demonstrated lower symptom severity compared to the control group, as indicated by the total score of all symptoms of 41.5 (29; 66) and 77 (41; 119), respectively (p < 0.05) (Table 2). Among these, the symptoms with significantly lower severity in the SCGJT group included cough, fatigue, hypogeusia, muscle aches, nasal congestion, runny nose, and sore throat (Figure 3). The utilization of Western drugs, including paracetamol, antibiotics, corticosteroids, anticoagulants, and antivirals, did not differ between groups (p > 0.05). However, the SCGJT group had lower demand for other symptom-relief medicines, such as cough and diarrhea, compared to the control group (p < 0.05) (Table 2).

**Table 2:** Study endpoints Note: Summary statistics are median (25th and 75th percentiles) or n (%). RT-PCR, reverse transcription-polymerase chain reaction; SCGJT, Shen Cao Gan Jiang Tang

Study endpoints	Control (N = 150)	SCGJT (N = 150)	p-value
Time to symptom resolution	13 (10; 16)	9 (6; 11)	<0.001
Total score of all symptoms	77 (41; 119)	41.5 (29; 66)	<0.001
Progression to severe COVID-19	7 (4.7%)	1 (0.7%)	0.067
Mortality	0 (0%)	0 (0%)	-
Days from randomization to discharge	8 (6; 11)	7 (6; 8)	<0.001
Paracetamol	61 (40.7%)	53 (35.3%)	0.405
Antibiotics	15 (10.0%)	17 (11.3%)	0.852
Corticosteroids	15 (10.0%)	14 (9.3%)	>0.999
Anticoagulants	12 (8.0%)	14 (9.3%)	0.838
Antivirals	9 (6.0%)	4 (2.7%)	0.256
Other symptom-relief medicines	72 (48.0%)	52 (34.9%)	0.026
Negative RT-PCR test on day 5	3 (2.0)	7 (4.7)	0.335
Negative RT-PCR test on day 7	29 (19.3)	60 (40.0)	<0.001
Negative RT-PCR test on day 10	101 (67.3)	121 (80.7)	0.012
Negative RT-PCR test on day 14	139 (92.7)	147 (98.0)	0.052
Time to viral clearance	9 (8; 12)	8 (7; 10)	<0.001

**Figure 4:**
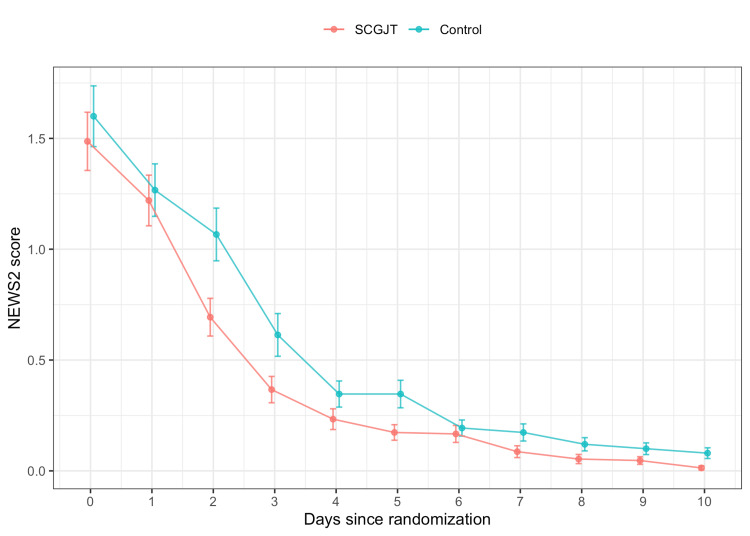
Daily NEWS2 score NEWS2, National Early Warning Score 2; SCGJT, Shen Cao Gan Jiang Tang

Time to discharge was shorter in the SCGJT group compared to the control group, with a median of 7 (6; 8) days and 8 (6; 11) days, respectively (p < 0.05) (Table 2). The NEWS2 score decreased over time, and there was no difference between groups (p > 0.05) (Figure 4). However, there were seven patients (4.7%) with severe disease in the control group, compared to only one (0.7%) in the SCGJT group, but this difference did not reach statistical significance (p > 0.05), and no deaths were reported (Table 2).

From the fifth day after randomization, RT-PCR was conducted and showed a decreasing trend in the proportion of SARS-CoV-2 infected individuals in both groups over time. The SCGJT group consistently had a higher rate of viral clearance, with all participants testing negative after 16 days from randomization, while the control group continued to have positive cases (p < 0.05) (Figure 2B). The time to viral clearance was shorter in the SCGJT group compared to the control group, with a median of 8 (7; 10) days and 9 (8; 12) days, respectively (p < 0.05) (Table 2).

No AEs were reported. The changes in routine lab tests before and after treatment in the two groups did not show differences when comparing the two groups (p > 0.05) (Table 3).

**Table 3:** Difference in routine lab tests from baseline to after day 10 Note: Summary statistics is mean ± SD. SCGJT, Shen Cao Gan Jiang Tang

Routine lab tests	Control (N = 150)	SCGJT (N = 150)	p-value
White blood cell count, ×10^9^/L	0.0 ± 2.9	-0.2 ± 2.8	0.337
Neutrophil count, ×10^9^/L	-0.1 ± 1.7	-0.1 ± 1.8	0.940
Lymphocyte count, ×10^9^/L	0.2 ± 1.3	-0.1 ± 1.4	0.236
Hemoglobin (g/dL)	-0.1 ± 1.3	0.1 ± 1.2	0.131
Platelet count, ×10^9^/L	-5.8 ± 78.3	-3.6 ± 82.1	0.912
Aspartate aminotransferase, U/L	0.5 ± 18.8	-0.6 ± 21.4	0.610
Alanine aminotransferase, U/L	0.0 ± 19.5	0.9 ± 20.1	0.532
Serum creatinine, umol/L	2.5 ± 26.0	2.3 ± 26.1	0.975
Fasting glucose (mmol/L)	-0.1 ± 0.9	0.0 ± 1.0	0.547

## Discussion

These findings indicated that SCGJT is effective in non-severe COVID-19 patients, as it reduces symptom duration and severity, including global severity and specific symptoms such as cough, fatigue, hypogeusia, muscle aches, nasal congestion, runny nose, and sore throat, as well as the need for medications and duration of hospitalization and infection. Although only one patient developed severe disease in the SCGJT group compared to seven in the control group, the difference was statistically insignificant. Furthermore, the NEWS2 score did not show a meaningful difference between groups. Therefore, conclusions regarding the preventive efficacy of SCGJT in disease progression remain inconclusive. No observed AEs were documented, and there were no significant alterations in routine test results, indicating the safety profile of SCGJT.

Since the onset of the COVID-19 pandemic, in the absence of specific treatments and the limited availability of vaccines, herbal medicines have gained widespread use, supported by substantial evidence of their effectiveness. As early as mid-2020, a rapid systematic review and meta-analysis by Fan et al., based on seven original studies, demonstrated that Chinese herbal medicines, when used as supplementary treatment alongside standard care, contributed to improved treatment outcomes in COVID-19 [16]. Furthermore, studies conducted across various countries and regions increasingly reveal diverse benefits associated with the use of herbal medicines in the management of COVID-19 [13,15,17-21]. In line with this trend, in Vietnam, herbal medicines have also been put into use immediately after the outbreak of the pandemic. Through this study, SCGJT, one of the Vietnamese herbal formulas, was demonstrated to be effective in treating non-severe COVID-19.

Previous studies consistently show that herbal medicines reduce symptoms, expedite symptom recovery, shorten virus nucleic acid positivity duration, decrease hospitalization time, alleviate progression to severe stages and mortality, and mitigate multi-organ damage in COVID-19 [22,23]. In general, the clinical effects appear quite similar across studies, whereas the impact of reducing the viral load of SARS-CoV-2 differs among various research findings. We attribute this discrepancy fundamentally to the use of different herbal medicine formulas. Although the viral load is believed to be unrelated to disease manifestations because the pathogenesis of the disease is associated with the complex interplay between the virus and the host’s immune response, reducing the viral load may lead to faster RT-PCR negativity, thereby decreasing the potential for transmission and reducing isolation and hospitalization time [14,24,25].

This study did not assess the mechanism of action of the medicinal formula. However, the effects can be explained based on the mechanism of action of the herbs present in SCGJT, as demonstrated in previous studies. Research on the SARS-CoV-2 virus noted that in some molecular docking and dynamics studies, *Radix Glycyrrhizae *has exhibited significant potential as an agent with antiviral, anti-inflammatory, and immunomodulatory properties [26]. Bioactive compounds found in ginger, specifically gingerol and shogaol, have demonstrated a notable affinity for the spike protein of the SARS-CoV-2 virus. This interaction suggests that these compounds can potentially interfere with the spike protein’s binding to the host angiotensin-converting enzyme 2. Such interference could potentially disrupt the viral entry process and subsequent infection [27]. Ginsenoside demonstrates broad-spectrum inhibitory activity against respiratory tract viruses, possesses immunomodulatory, antioxidant, and anti-inflammatory properties, and shows potential for targeting n-CoV-2 proteins, including SARS-CoV-2 and influenza A [28]. Nevertheless, whether combining all these herbs into one formula alters the mechanism of action remains unknown.

According to TM, COVID-19 is considered a disease caused by the unauthorized invasion of external pathogenic factors into the body [5,23]. The stronger external pathogenics and the weaker Qi, the more likely the disease is to penetrate deeply and become more severe [5,23]. Generally, many herbal formulas have been studied, all following common principles like Qi enhancement and expulsion of external pathogenics [5,29]. However, external pathogenics in different cases may manifest distinct characteristics, including cold-damp, damp-heat, and damp-toxin, even when at the same disease stage [23]. Therefore, to be applicable across a diverse range of patients with varying TM syndromes, the SCGJT formula has been selected for its general efficacy in enhancing Yang Qi to resist external pathogens. This is particularly beneficial in the case of non-severe COVID-19 patients when the Qi in the body is still robust. Remarkably, the growing number of SARS-CoV-2 variants presents varying symptoms and severity, resulting in diverse diagnoses of external pathogenics and corresponding distinctions in TM treatments [23,30]. This supports a preference for formulas that enhance Yang Qi rather than tailoring treatments for external pathogens when broadly applied without a detailed TM syndrome diagnosis. However, further research is needed to determine the most effective treatment option for the community.

This study has several limitations. Firstly, all patients received only a single dose of the vaccine, whereas full-dose vaccination is now widely accessible, which may limit generalizability. Secondly, the study did not employ blinding in the assignment of interventions, potentially leading to bias in outcome assessment. Thirdly, various SARS-CoV-2 variants have emerged to date; thus, further research is needed to explore the effectiveness of SCGJT.

## Conclusions

SCGJT, a Vietnamese herbal formula aimed at enhancing Yang Qi, demonstrated clinical efficacy in non-severe COVID-19 patients and showed safety data when combined with standard care. The duration of SARS-CoV-2 viral clearance also appears to be shorter when using SCGJT, while data on preventing severe progression is still inconclusive. Furthermore, given the changing landscape of the COVID-19 pandemic, the effectiveness of SCGJT needs to be rigorously monitored for further insights into these questions.
